# Potential role of marine species-derived bioactive agents in the management of SARS-CoV-2 infection

**DOI:** 10.2217/fmb-2021-0024

**Published:** 2021-10-25

**Authors:** Muhammad Asif, Mohammad Saleem, Hafiza Sidra Yaseen, Ashwaq HS Yehya, Malik Saadullah, Hafiz Muhammad Zubair, Chern E Oon, Pegah Moradi Khaniabadi, Syed Haroon Khalid, Ikram Ullah Khan, Mahrukh

**Affiliations:** 1 Department of Pharmacology, Faculty of Pharmacy, The Islamia University of Bahawalpur, Bahawalpur, 63100, Punjab, Pakistan; 2 Punjab University College of Pharmacy, University of the Punjab, Lahore, 54000, Punjab, Pakistan; 3 Faculty of Pharmaceutical Sciences, Government College University Faisalabad, Faisalabad, 38000, Punjab, Pakistan; 4 Institute for Research in Molecular Medicine (INFORMM), Universiti Sains Malaysia, Penang, 11800, Malaysia; 5 Department of Radiology & Molecular Imaging, College of Medicine & Health Sciences, Sultan Qaboos University, PO. Box 35, 123, Al Khod, Muscat, Oman

**Keywords:** anti-inflammatory, COVID-19, fingolimod, griffithsin, marine drugs, plitidepsin, SARS-CoV-2

## Abstract

COVID-19, caused by the SARS-CoV-2 outbreak, has resulted in a massive global health crisis. Bioactive molecules extracted or synthesized using starting material obtained from marine species, including griffithsin, plitidepsin and fingolimod are in clinical trials to evaluate their anti-SARS-CoV-2 and anti-HIV efficacies. The current review highlights the anti-SARS-CoV-2 potential of marine-derived phytochemicals explored using *in silico*, *in vitro* and *in vivo* models. The current literature suggests that these molecules have the potential to bind with various key drug targets of SARS-CoV-2. In addition, many of these agents have anti-inflammatory and immunomodulatory potentials and thus could play a role in the attenuation of COVID-19 complications. Overall, these agents may play a role in the management of COVID-19, but further preclinical and clinical studies are still required to establish their role in the mitigation of the current viral pandemic.

## Graphical abstract

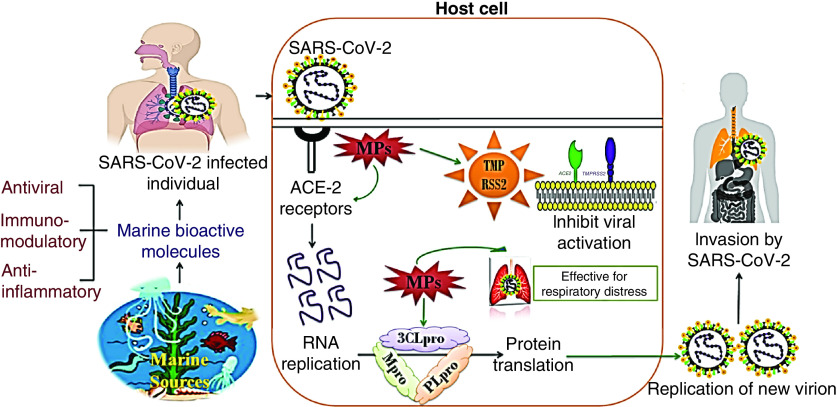

Chronic respiratory diseases are among the most prevalent secondary conditions in SARS-CoV-2-infected patients [1]. The rapidly changing world is halted now because of the COVID-19 pandemic, which has affected almost 212 nations on seven continents around the globe. SARS-CoV-2 is an enveloped positive-stranded RNA virus and belongs to the β genus of the family Coronaviridae [2]. Individuals living in nursing homes who are elderly (>50 years) and have underlying chronic pathologies (diabetes, cardiovascular ailments and immunocompromised conditions) are at greater risk of contracting COVID-19. Complications of COVID-19 may mimic other chronic pathologies such as viral pneumonia, chronic respiratory disease and cardiac abnormalities [3]. The viral spike proteins (S-proteins) and the enzymes including dipeptidyl peptidase (DPP4) and aminopeptidase N (APN) are known to contribute in the SARS-CoV-2 pathogenicity by facilitating its entry into human lung cells. Following attachment, the virus takes over the host cell’s machinery to translate RNA into proteins. This process is activated through proteases that play a critical role in viral propagation [4]. Genomic characterization of SARS-CoV-2 suggests that the Malayan pangolin (*Manis javanica*) and Chinese bats (*Rhinolophus sinicus*) are the likely genetic sources of CoV-β viruses and are capable of infecting human beings [5]. The genome of CoVs contains a primary protease (Mpro) and papain-like proteases (PLpro) as basic polyproteins that are considered to be the targets of vaccines [6]. Data collected from WHO vaccine tracker shows that seven vaccines are approved by WHO for human use against COVID-19 [7] and several are currently in the different phases of clinical trials or in the queue for human clinical trial approvals [8]. Well known international research institutes including CanSino Biologics Inc. (Tianjin, China), Beijing Institute of Biotechnology (Beijing, China), University of Oxford (Oxford, UK), Inovio Pharmaceuticals (CA, USA), Sinovac Biotech Ltd. (Beijing, China), Wuhan Institute of Biological Products (Zhengdian Jiangxia District Wuhan, China), Pfizer-BioNTech (USA and Germany) and Moderna TX, Inc. (MA, USA) have been hot on the heels to develop the world’s first effective vaccine against COVID-19 [9]. According to Nature news reports, some of these vaccines, including Moderna’s vaccine has shown more than 94% efficacy in preliminary human trials. However, these data need to be peer reviewed [10]. In the past two decades, deaths caused by SARS and MERS pandemics have highlighted the pathological potentials of coronaviruses and indicate the need for further development of antiviral therapies including anti-SARS-CoV agents [11].

Natural products (antibiotics, marine compounds and phytochemicals) have been the main sources for the development of innovative pharmacological agents. It is estimated that around one third of the marketed drug products are derivatives of natural products, either directly or indirectly [12]. During the last two decades, the consumption of natural products has been greatly reduced due to difficulty in their practical explorations. However, the evolution of modern cultivation and extraction technologies has revived the medicinal value of natural products [13]. Marine biomass like bacteria, viruses, archaea, fungi and algae comprise almost 70% of sea life and overall one third of the planet [14]. Over the past few decades, marine plants and microbes have been the focal point of scientific research due to their characteristic biological properties. More than 12,000 natural products have been obtained from marine plants and microorganisms, and this list is continuously expanding [15].

Increasing evidence has highlighted the therapeutic potential of marine-derived moieties in the identification of novel templates/prototypes against COVID-19 [16]. Drugs used as anti-COVID-19 therapies can perform their functions through two processes, drugs targeting SARS-CoV-2 viruses directly or drugs targeting host cell proteins. The genome of SARS-CoV-2 comprises four structural proteins, including spike glycoproteins (S), matrix glycoproteins (M), nucleocapsid proteins (N) and small envelope proteins (E). Moreover, the main protease (MPro) and 3CLpro, which mediates the transcription, replication and maturation of coronaviruses, are also targets of anti-SARS-CoV-2 therapy. Advances in bioinformatics and computational modeling are great aids in screening repurposed drugs and marine bioactive products for their potential to block various steps involved in SARS-CoV-2 entry and propagation in human cells and the resultant respiratory complications [17].

A plethora of scientific evidence highlights the therapeutic potential of bioactive compounds obtained from different marine organisms. Ziconotide (Prialt^®^) is a synthetic compound obtained from the toxin of cone snails (*Conus magus*) and has been used for AIDS and cancer-associated chronic pain. Moreover, clinical trials have shown that Prialt^®^ acts as a non-narcotic pain-relieving agent and thus, can be used as an alternative to morphine [18]. Tunicates (sea squirts) are a rich source of active biopharmaceuticals. Didemnin B, an antiviral agent extracted from *Trididemunm solidum*, has shown strong antiviral potential against DNA and RNA viruses [19]. *Ecteinascidia turbinata*-derived compound, trabectedin (Youndelis^®^) has been accepted as an antineoplastic agent in the European Union (EU) [20]. Eribulin mesylate marked with the brand name of Halaven^®^ is a synthetic analogue of halichondrin B which is a marine sponge (*Halichondria okadai*)-derived macrolide. It is known to have potent anticancer properties in metastatic breast cancer patients [21]. The utilization of marine organism-derived bioactive compounds as prototypes for the development of COVID-19 multi-targeted therapy is the need of the time. A molecular dynamic (MD) simulation study conducted by Khan and colleagues described the inhibitory effects of terpenoid compound (T3) obtained from the marine sponge (*Cacospongia mycofijiensis*) toward the SARS-CoV-2 Mpro enzyme. Data from this study highlighted the high binding affinity and low flexibility of T3, which can act as an excellent candidate against SARS-CoV-2 [22]. Plitidepsin (Aplidin^®^) is a marine-derived (*Aplidium albicans*) drug with anticancer and antiviral properties. A recent *in vitro* study conducted by White *et al.* showed the potent anti-SARS-CoV-2 potential of plitidepsin in two cell lines. It was found to be more potent than remdesivir in inhibiting viral replication in a human cell line (hACE2-293T) [23]. Plitidepsin has also been tested in Phase I/II clinical trials to test its efficacy in COVID-19 patients. Although the results are not yet published, it is suggested by the manufacturer that this agent should be strongly considered for expanded clinical trials to treat COVID-19 patients [23]. In addition to these, other studies have reported the therapeutic potential of natural products, including marine-derived compounds, against SARS-CoV-2 infection [2,24,25]. Data collected from web show that, from 1969 to 2020, a total of 15 marine-derived drugs were approved by the US FDA for the treatment of various chronic disorders including cancers, severe pain and herpes simplex viral infections [26]. Table 1 lists the pharmacological attributes of selected bioactive molecules obtained from different marine species.

**Table 1.** Marine species-derived bioactive compounds in various phases of clinical evaluations.

Compound	Source	Clinical manifestations and status	Mechanism of action	Ref.
Ecteinascidin 743	*Ecteinascidia turbinate* (ascidian)	In Phase II clinical trials to evaluate its efficacy in adult patients with advanced soft-tissue sarcoma (identifier: NCT02359474), meningioma (identifier: NCT02234050), breast neoplasms (identifier: NCT00580112), ovarian cancer (OC) (identifier: NCT00050414) and other neoplastic disorders	Interacts with minor grooves of DNA and interferes with cell division, DNA transcription and repair mechanisms	[27,28]
Bryostatin 1	*Bugula neritina* (brayozoan)	In Phase II clinical trials for progressive kidney cancer (identifier: NCT00005056), colorectal cancer (identifier: NCT00003220) and other neoplastic disorders	Downregulates protein kinase-C signaling pathway ultimately inhibiting cell differentiation. It can also cause T-cells activation	[29]
IPL-576,092	*Petrosia contignata* (sponge)	In Phase II clinical trials for asthma-associated problems	Inhibits activation of inflammasomes, e.g., interleukins, histamine, NF-kß	[29,30]
Cematodin (synthetic derivative of Dolastatin 15)	*Dolabella auricularia* (mollusk)	In Phase II clinical trials as an anticancer agent	Interruption of microtubular network of cancer cells	[28]
Didemnin B	*Trididemunm solidum* (sea squirts)	In Phase I clinical trials as an anti-inflammatory agent	Inactivation and blockage of inflammatory mediators e.g., serotonin, histamine, TNF-α	[31]
Pseudopterosins	*Pseudopterogorgia elisabethae* (Carribean soft coral)	In advanced preclinical trials as an anti-inflammatory and analgesic agent	-------------	[27,29,32]
Halichondrin B source of eribulin mesylate a halichondrin B analog, marketed as Halaven®	*Lissodndoryx* species (deep water sponge metabolite)	Approved for breast cancer treatment. In Phase II clinical trials for recurrent breast carcinoma and stage IV breast cancer (identifier: NCT01908101), hormone-refractory prostate cancer (identifier: NCT00337077), solitary fibrous tumor (identifier: NCT03840772)	Microtubule-depolymerizing agent	[33]
Discodermolide (XAA296A)	*Discodermia* genus (deep water sponge metabolite)	In Phase I clinical trials as an anticancer agent	Interruption of a microtubular network of cancer cells	[34]
Vidarabine, Marketed as Vira-A^®^	*Tethya crypta* (Caribbean sponge)	Herpes encephalitis, acyclovir-resistant HSV and VZV	1. Inhibitor of viral DNA synthesis. 2. Incorporation into viral RNA as well as DNA	[35]
PM01183 (PM; lurbinectedin)	Ascidian	Anticancer properties in Phase III clinical trial (identifier: NCT02421588)	Blocks *trans*-activated transcription induces DNA double-strand breaks, and inhibits the tumor microenvironment	[36,37]
Plinabulin (NPI-2358, a synthetic analog of the diketopiperazine halimide)	*Aspergillus* sp. (marine fungi)	In Phase III clinical trial (identifier: NCT03294577)	Plinabulin inhibits tubulin polymerization, leading to the disruption of the vascular endothelial architecture of the tumor	[38–40]
Tisotumab vedotin (TV)	Isolated from mollusks and *cyanobacteria*	In Phase II clinical trial (identifier: NCT03657043)	Tisotumab vedotin (TV) is a first-in-class antibody-drug conjugate comprising a TF-targeted fully human monoclonal antibody conjugated to the microtubule-disrupting agent monomethyl auristatin E	[39,41]
Sodium oligomannate (GV-971)	Marine-derived Oligosaccharide, a mixture of linear and acidic oligosaccharides	For mild-to-moderate Alzheimer’s dementia in Phase III clinical trial (identifier: NCT04520412)	It can reconstitute the gut microbiota, reduce bacterial metabolite-driven peripheral infiltration of immune cells into the brain, and inhibit neuro-inflammation in the brain as observed in animal models. Some GV-971 can penetrate the brain and directly inhibit Aβ fibril formation and destabilizes the preformed fibrils into nontoxic monomers	[42]
ET-743	*Ecteinascidia turbinate* (colonial tunicate)	Antitumor activity in patients with advanced resistant sarcoma in Phase III clinical trial (identifier: NCT02903004)	1. Induces a broad inhibition of activated transcription with no effect on the constitutive transcription. 2. ET-743 inhibits the activation of the multidrug-resistant pathway.	[43]
Plitidepsin	*Aplidium albicans* (mediterranean tunicate)	Antitumor evaluation against solid tumors and lymphoma in Phase II clinical trial (identifier: NCT01149681)	Induces cytotoxicity in a non-MDR/p53 dependent manner, blocks the cell cycle progression at G1, and decreases the secretion of the VEGF and the expression of the VEGF-r1 receptor	[43,44]
		Relapsed/refractory non-Hodgkin’s lymphoma in Phase II clinical trial (identifier: NCT00884286)	Plitidepsin has shown activity against several human malignant cell lines, including leukemias and lymphoma	[45].
Marizomib (NPI-0052; salinosporamide A)	Marine actinomycete	Multiple myeloma, lymphomas, leukemias and solid tumors Phase I clinical trial (identifier: NCT00629473)	Third-generation proteasome inhibitor that irreversibly binds to all three subunits (b1 [caspase-like; C-L], b2 [trypsin-like; T-L] and b5 [chymotrypsinlike; CT-L]) of the 20S proteasome	[46]
Squalamine lactate (MSI-1256F)	*Squalus* *acanthias* (stomach of shark)	Ophthalmic problems involving pathological angiogenesis in Phase II and III clinical trials (identifier: NCT01678963)	1. Antibiotic activity 2. Angiogenesis inhibitor	[38]
Tetrodotoxin (TTX)	Fish, algae and bacteria	Peripheral-acting analgesia for chronic pain in Phase I clinical trial (identifier: NCT04083833)	Highly specific VGSC blocker in clinical evaluation	[47,48]
3-(2,4-Dimethoxybenzylidene)-anabaseine (also known as GTS-21 or DMXBA)	Synthetic alkaloid derivative of anabaseine obtained from marine nemertine	In the clinical trials for anti-inflammatory effects. 1. Phase I/II for attention deficit hyperactivity disorder. 2. Phase II for schizophrenia 3. Phase II for Alzheimer’s disease (identifier: NCT00100165)	1. Preferentially stimulate α7 nicotinic choline receptors. 2. GTS-21 inhibits the production of pro-inflammatory cytokines through its effects on α7 nicotinic acetylcholine receptors on the macrophage	[49]
Dolastatin-10	*D. auricularia* (marine shell-less mollusc)	The cytotoxic peptide in Phase II clinical trial (identifier: NCT00003626)	Microtubule inhibition, BCL-2 phosphorylation and apoptosis induction	[50]

Only datum of clinical trials which were completed or in the phases of recruitment is included.

OC: Ovarian cancer; VGSC: Voltage-gated sodium channel; VZV: Varicella Zoster virus.

## Search methodology

Relevant information was collected from review articles, web contents, clinical reports and research papers available at numerous databases including PubMed, ScienceDirect, SciFinder, Google Scholar, Clinicaltrails.gov, ProQuest, Medline and Scopus, respectively. Moreover, the global marine pharmaceutical pipeline website (https://www.marinepharmacology.org/) maintained by Alejandro MS Mayer at Midwestern University, IL, USA was also consulted to track the current status of marine species-derived bioactive molecules [51]. To locate authentic information from published records, the following combination of terms were used: ‘coronavirus and natural drugs’, ‘microbial metabolites’, ‘marine by-products against SARS-CoV-2’, ‘synthetic products of marine origin’, ‘marine drugs’, ‘sea-life compounds’, ‘marine reservoirs having therapeutic potential’, ‘SARS-CoV-2 and marine species-derived agents’ and ‘natural products and COVID-19’. The studies encompassing information relevant to crude extracts, fractions, isolated compounds and secondary metabolites obtained from marine species and acting against SARS-CoV-2 were included. All other information not fulfilling the inclusion criteria, duplicate data and having approved antiviral effects other than COVID-19, were excluded.

## Results & discussion

Medicinal agents obtained from marine organisms are known to have antiviral activities against enveloped viruses, including coronaviruses (CoVs) [52]. Marine sourced drugs may get access to the market through sustainable availability of an adequate number of organisms without affecting the marine environment [53]. Recently, the USA and Japan have launched new programs for the successful cultivation of sea-life (bacteria, fungi and so on) which are now becoming a potential source of novel and life-saving bioactive agents [54] with profound anti-inflammatory, anticancer and antimicrobial properties. In addition, the development of a universal expression system for the synthesis of small molecules and constructing biogenetic tools for the *in vivo* evaluation of cultured marine microorganisms are also basic requirements for this research. This review highlights the key findings of various scientific studies conducted to explore the antiviral activities (especially anti-SARS-CoV-2) of medicinal products obtained from different marine organisms (bacteria, cyanobacteria, fungi, algae and invertebrates).

### Alkaloids

Quimque and colleagues used molecular docking and MD simulation tools to predict the binding affinity of two marine alkaloids obtained from fungi i.e., scedapin C and norquinadoline A, against major targets of SARS-CoV-2. Both compounds showed the highest binding affinity against PLpro with binding energy values of -10.9 kcal/mol [55]. PLpro has been known to play a major role in SARS-CoV-2 replication within host cells and the deactivation of host immune responses by its interaction with ubiquitin protein [56]. Ubiquitin protein is associated with the activation of NF-κβ signaling with subsequent production of IFN-β in the host [57]. IFN-β is known to have antiviral properties against many viruses, including SARS-CoV-2 [58]. Upon entry of SARS-CoV-2 into the host cell, it releases PLpro, which serves two functions: first, these proteases help in the replication of viruses in the host cell [59], and secondly, they deactivate host immune responses by suppressing NF-κβ activation and subsequently inhibit IFN-β production [57]. Therefore, based on the findings of this molecular simulation study it can be proposed that scedapin C and norquinadoline A alkaloids have ability to inhibit SARS-CoV-2 PLpro, thus blocking viral replication with simultaneous activation of host immune responses through NF-κβ signaling pathway. Further detailed *in vitro* and *in vivo* studies are warranted in this regard to support this hypothesis.

A recent study conducted by Khan and colleagues demonstrated the efficacy of five marine alkaloids i.e., C1 (fostularin-3) and C2 (chimyl alcohol) obtained from *Aplysinidae* family, C3 (palmitoleic acid) obtained from *Pterogorgia citrine*, and C4 (cannabigerolic acid) and C5 (acitretin) obtained from *Petrosia strongylophora* sp. against SARS-CoV-2. Data of molecular docking and MD simulation techniques exhibited that fostularin-3 (C1) formed hydrogen bonds and hydrophobic interactions with Thr24, Leu27, His41, Phe140, Cys145, His163, Met165, Pro168 and His172 residues in the active site of the M^pro^ enzyme of SARS-CoV2 [22].

Caulerpin is another alkaloid obtained from different species of marine algae including green macroalgae (*Caulerpa racemose*), red algae (*Chondria armata*), and brown algae (*Sargassum platycarpum*). Abdelrheem and colleagues used molecular docking and MD simulation techniques to predict the effects of caulerpin against the main protease of SARS-CoV-2 (3CLpro) and its pharmacokinetic properties. The results of the molecular docking study indicated the strong binding affinity of caulerpin with 3CLpro. Moreover, it was found to form a stable complex with 3CLpro in explicit water. This complex had no major effect on the flexibility of the protein throughout the simulations, making it a suitable candidate in COVID-19 treatment [60]. Besides this, caulerpin has been reported to have well-known potential against pathogenic viral strains like HSV-1 and hRSV [61]. 3CLpro is known to play a major role in the maturation of virus [60] so keeping in mind the data of the above mentioned computer simulation study, it can be concluded that caulerpin has the potential to halt the SARS-CoV-2 life cycle. Various studies have reported the *in vivo* anti-inflammatory properties of caulerpin. Data from these studies revealed that it produces anti-inflammatory effects through the down-regulation of TNF-α, IFN-γ, IL-6 and IL-17 [62,63]. Studies have shown that TNF-α and IL-6 are major culprits secreted in excess during cytokine storm phase (hyperinflammatory responses) of COVID-19 and are responsible for organ damage [64,65]. IL-6 is known to stimulate the secretion of IL-17, another pro-inflammatory cytokine, from Th17 cells via the JAK2/STAT3 pathway. On the basis of these studies, it is proposed that caulerpin, which has favorable pharmacokinetic (ADMET) properties, has the potential to not only block SARS-CoV-2 3CLpro actions but also can attenuate inflammation via the down-regulation of multiple pro-inflammatory cytokines released during the hyperinflammatory phase of COVID-19.

### C-phycocyanin

C-phycocyanin is a protein-bound pigment of the blue-green algae (*Arthrospira platensis*), commonly known as spirulina. Raj and colleagues screened its binding affinity with the non-structural proteins (nsp-8, nsp-7 and nsp-12) of SARS-CoV-2. These proteins, particularly nsp-12, are known to play a major role in SARS-CoV-2 multiplication in the host cells. Docking data revealed that C-phycocyanin has the potential to inhibit the active sites of nsp-12 [66]. Moreover, spirulina is used as a food supplement due to its rich nutritional composition including vitamin C and minerals. Studies have suggested that elderly people should consume spirulina in order to boost their immunity against SARS-CoV-2 [67]. Moreover, spirulina is known to have anti-inflammatory properties *in vivo* through the inhibition of inflammatory cells recruitment and down-regulation of TNF-α and nitric oxide release [68]. Keeping in view the demonstrated pharmacological properties of spirulina, it is urged that it should be studied for its effectiveness in the management of various phases of COVID-19 using well-planned preclinical and clinical studies.

### Polyphenols

Studies have shown that various algae polyphenols (quercetin and its analogues) derived from the brown macro algae of genus *Sargassum*, possess potent antiviral properties [69]. Data obtained from a scientific report showed that quercetin plays a significant role in the deactivation of interaction sites of SARS-CoV-2 proteins that attack human ACE2 receptors in the lungs. Quercetin showed low binding energy and extraordinary potency for the targeted proteins, and thus can be considered as a potential agent in the development of anti-SARS-CoV-2 therapy. However, further animal studies are suggested to confirm the efficacy of quercetin as an anti-CoV therapy [14]. SARS-CoV-2 M^pro^ is an enzyme with a dominant function in viral replication and translation thus, can be considered an attractive target site for anti-CoV drugs [70]. Based on the extensive ongoing investigations on marine-derived flavonoids, Antonio and colleagues studied the M^pro^ inhibitory effects of quercetin, apigenin, luteolin and amentoflavone (bioflavonoids) in *in vitro* assays. They revealed that poly-hydroxylated mixtures impart a prominent role in the development of M^pro^ noncompetitive inhibitors, and exhibit marked potential against SARS-CoV-2 reproduction. A few studies have shown that quercetin-based nebulization alleviates respiratory symptoms [71]. Recruitment of monocytes and macrophages in COVID-19 leads to lung inflammation and pulmonary fibrosis [72]. Hence, quercetin isolated from an algal (*Acanthophora spicifera*) species was tested extensively for its pharmacological actions against respiratory pathology [73]. Multiple studies have shown that almost all COVID-19-infected patients have lungs abnormalities due to the release of inflammatory mediators, thus, it is suggested that quercetin may serve as an attractive alternative therapy for inflammation-associated respiratory ailments, including COVID-19 [74]. Several *in vivo* and *ex vivo* studies have reported the virucidal effects of quercetin *via* interruption of the entry and attachment phases of the viral life cycle (e.g., HCV, herpes simplex virus, hepatitis B virus, vesicular stomatitis virus, adenovirus and influenza-A virus). Molecular docking techniques and *ex vivo* as well as *in vivo* models have provided the basis to explore quercetin’s potential against SARS-CoV-2 [75]. Findings of the recent pilot randomized controlled trial showed that treatment of COVID-19 patients with Quercetin Phytosome^®^ resulted in the faster recovery and marked reduction in blood parameters i.e., LDH (-35.5%), Ferritin (-40%), CRP (-54.8%) and D-dimer (-11.9%) [76]. However, the emergence of high-quality clinical data is encouraged to support the possible efficacy of quercetin in the context of the recent viral pandemic (SARS-CoV-2) [77].

## Sulfated polysaccharides

Song and colleagues studied the *in vitro* inhibitory activities of four marine sulfated polysaccharides, i.e., sea cucumber sulfated polysaccharide (SCSP), fucoidan from brown algae, iota-carrageenan from red algae and chondroitin sulfate C from sharks (CS) against SARS-CoV-2. Among these polysaccharides, SCSP, fucoidan and carrageenan showed significant anti-SARS-COV-2 activities at the concentrations of 3.90–500 μg/ml. SCSP exhibited the strongest inhibitory activity among these compounds. Further experimentation using pseudo-type virus bearing the S-glycoprotein showed that SCSP can bind to the S-glycoprotein of SARS-CoV-2 thus, indicating the potential to prevent SARS-CoV-2 entry into the host cells. Based on the findings of this *in vitro* study, it was suggested that sulfated polysaccharides obtained from different marine organisms can be employed to treat and prevent COVID-19 [78].

Mechanical ventilation with oxygen is widely used to treat various lung illnesses; however, it may result in hyperoxia, causing lung inflammation and injury [79]. *In vivo* studies conducted by Nie and colleagues showed that treatment with fucoidan prevented lung injury by reducing hyperoxia-induced inflammation in mice and suppressing the expression of pro-inflammatory cytokines (IL-1, IL-16 and TNF-α) *via* the ERK signaling pathway [79]. A review by Fitton and colleagues highlighted that fucoidan has immune boosting properties. Moreover, it has potent vaccine adjunct properties [80] and studies conducted by Negishi *et al.* in elderly Japanese patients showed that oral administration of a fucoidan extract enhanced their responses to influenza vaccines [81]. Taken together, these findings highlight that fucoidan not only has the potential to target SARS-CoV-2, but can also produce beneficial effects in humans by increasing their responsiveness to the COVID-19 vaccines as well as reducing serum levels of pro-inflammatory cytokines. However, further pre- and clinical studies in this regard are warranted.

### Briarane-type diterpene

A molecular docking study calculated the binding potency of natural products obtained from a variety of sources including marine organisms against major drug targets of SARS-CoV-2. The data showed that excavatolide M, a briarane-type diterpene obtained from gorgonian (*Briareum excavatum*), has the potential to bind and block SARS-CoV-2 TMPRSS2 (S-protein) [14]. However, another docking study conducted by Rahman and colleagues predicted the toxic nature of excavatolide M [82]. Further studies to explore the pharmacological and toxicological profile of excavatolide M are required to establish its safe dose and exact mechanism of action.

### Sesquiterpene quinone

A study by Surti and colleagues evaluated the potency of illimaquinone, a marine sponge metabolite first identified and isolated from brown sponge named *Hippospongia metachromia*, against SARS-CoV-2 using *in silico* approach broadly. Efficacy of ilimaquinone against nine potential SARS-CoV-2 target proteins was calculated and compared with hydroxychloroquine, azithromycin, favipiravir, ivermectin and remdesivir using molecular docking studies. Binding energies data showed that ilimaquinone hold promising inhibitory potential against all the SARS-CoV-2 target proteins. The study concluded that ilimaquinone can be the most promising inhibitory candidate against the SARS-CoV-2 papain like protease [83].

### Fingolimod

Fingolimod is a synthetic compound that was synthesized from myriocin, a metabolite of the fungus called *Saria sinclairii*. The Australian and American drug regulatory authorities have approved the use of fingolimod in relapsing-remitting multiple sclerosis, an autoimmune disease [84]. Data on the use of fingolimod in COVID-19 patients is still not clear. One group of scientists suggests that it could be beneficial in COVID-19 patients as it can arrest the invasion of lymphocytes into alveoli [85], which could be beneficial as further lung damage can be prevented. Another group of scientists reported clinical improvement in COVID-19 patients with multiple sclerosis [86]. Moreover, a third group of scientists reported worsening of SARS-CoV-2 infection in multiple sclerosis patients upon the discontinuation of fingolimod therapy [87]. A clinical trial registered at https://clinicaltrials.gov (identifier: NCT04280588) is on the way (Phase II) to establish the efficacy of fingolimod in COVID-19 patients.

### Carboxylic acid ethyl ester

An *in silico* study by Vijayaraj and colleagues tested the binding affinities of eight marine organism-derived compounds, i.e., glycosaminoglycan, polyacetylenetriol, dehydrofurodendin, esculetin ethyl ester, dolabelladienetriol, arabinofuranosyl hypoxanthine (ara-H), 3-hydroxyleucine and hamigeran B against the SARS-CoV-2 protease N3. Docking results showed strong binding interactions of esculetin ethyl ester, obtained from *Axinella* cf.* corrugata* (marine sponge) with the viral protease [88].

### Seaweed lectins

Seaweed lectins, especially highly mannose-specific lectins from red algae including griffithsin derived from *Griffithsia* sp., are known to block the replication of various enveloped viruses like HIV-1, influenza and herpes viruses in *in vitro* assays. In 2010, O’Keefe and colleagues reported that griffithsin binds to the SARS-CoV spike glycoprotein and inhibits viral entry into host cells. Moreover, it prevented damage to host cells caused by the abnormal activation of the host immune system during the infection [89]. Millet *et al.* in 2016 reported the antiviral activity of griffithsin against another strain of coronaviruses, MERS-CoV. It was reported to inhibit the entry of MERS-CoV into host cells by blocking its spike proteins [90]. Barre and colleagues conducted a detailed *in silico* docking study to explore the potential anti-SARS-CoV-2 properties of griffithsin. It was proposed to hamper the attachment of SARS-CoV-2 to host cells by making a complex with S-proteins [25]. Data from https://clinicaltrials.gov (identifiers: NCT02875119 and NCT04032717) show that two clinical trials are registered to evaluate the efficacy of griffithsin in HIV infections. This indicates that this glycoprotein has the potential to be developed as an antiviral or an anti-SARS-CoV-2 nutraceutical. Table 2 highlights the summary of selected marine bioactive molecules against COVID-19.

**Table 2.** Mechanisms, specific functions and characteristics of selected marine-derived natural products against severe acute respiratory syndrome coronavirus.

Drugs	Source	Mechanism of action	Specific characteristics	Ref.
Scedapin C and norquinadoline A	*Scedosporium apiospermum* (Fungi)	Having the ability to bind with putative binding sites of PLpro through H-bonding and inhibit SARS-CoV-2 activation	Antiviral and protease inhibitor	[55,56]
Fostularin-3 and chimyl alcohol	*Alpysinidae*	Have the potential to bind with residues of active sites of M^pro^ of SARS-CoV-2	Antiviral and protease inhibitor	[22]
Iota-carrageenan	Red algae	Binds with S-glycoprotein of SARS-CoV-2 and stops the entry of virus in the host cell	Antiviral and inhibitors of proliferative proteins	[60]
Fucoidan	Brown algae	Blocks ERK signaling pathway and inhibits S-glycoprotein of SARS-CoV-2	Antiviral, immunomodulatory and anti-inflammatory	[78]
Palmitoleic acid	*Pterogorgia citrine*	Blocks M^pro^ activation and counter endothelial dysfunctioning to inhibit inflammatory pathways	Antiviral, anti-inflammatory and protease inhibitor	[22]
Cannabigerolic acid and Acitretin	*Petrosia strongylophora*	Targets essential proteins involved in the lifecycle of SARS-CoV-2 through deactivation of active sites of M^pro^	COX-enzyme inhibitor and antiviral	[22]
C-phycocyanin	*Arthrospira platensis* (blue-green algae)	Acts on active sites of nsp-12 and inhibit the multiplication of SARS-CoV-2	Immunomodulatory, anti-inflammatory, antiviral and nutritional supplement	[67,68]
Caulerpin	*Caulerpa racemosa*	Have significant binding affinity with 3CLpro to stop SARS-CoV-2 activation and attenuate inflammation via down-regulation of IL-6, TNF-α etc.	Anti-inflammatory and antiviral	[64,65]
Quercetin and derivatives	*Sargassum* genus	Causes deactivation of interaction sites of SARS-CoV-2 proteins on ACE-2 receptors and exerts M^pro^ noncompetitive inhibition	Anti-inflammatory and antiviral	[68]
Excavatolide M	*Gorgonian briareum excavatum*	Blocks SARS-CoV-2 TMPRSS2	Antiviral	[91]
Esculetin-4-carboxylic acid ethyl ester	*Axinella corrugate* (marine sponge)	Significantly inhibits binding affinity of SARS-CoV-2 with 3CLpro	Antiviral	[83]
Griffithsin	*Griffithsia* sp.	Binds with SARS-CoV-2 spike glycoprotein thus inhibited viral entry into the host cells	Immunomodulatory and antiviral	[88,89]
Fingolimod (a synthetic compound)	*Isaria sinclairii* (fungus)	Arrests the invasion of lymphocytes in alveoli and blocks active site of SARS-CoV-2	Immuno-protective, antiviral	[86]

PLpro: Papain-like protease; SARS-CoV-2: Severe acute respiratory syndrome coronavirus.

## Miscellaneous agents

Gentile and colleagues used *in silico* docking tools to explore the anti-SARS-CoV-2 potential of natural products obtained from different marine sources. Out of 770 compounds that met the pharmacophore criteria, 17 compounds including heptafuhalol A, phlorethopentafuhalol A and B, pseudopentafuhalol C, pseudotheonamide C and D, hydroxypentafuhalol A, pentaphlorethol B, 6,6’-Bieckol, 8,8’-Bieckol, apigenin-7-oneohesperidoside, luteolin-7-rutinoside, dieckol, aeruginosin 98B, resinoside B, pentaphlorethol A and tunichrome An2 were found to have promising SARS-CoV-2 protease inhibitory potentials [92]. Further *in vitro* and *in vivo* studies to establish the possible role of these agents in the prevention and treatment of SARS-CoV-2 infections and associated complications are warranted.

## Conclusion

In the current health emergency, COVID-19 has become a global issue affecting the daily routine of millions of people around the globe. Data obtained from currently available studies point towards promising anti-SARS-CoV-2 potential of medicinal agents obtained from various marine organisms. Some of these like spirulina are already in human use as a food supplement and other like fingolimod, griffithsin and plitidepsin are under clinical trials to evaluate their anti-SARS-CoV-2 and anti-HIV efficacies.

## Limitations & future perspective

Currently available studies on the use of marine-derived medicinal agents as anti-SARS-CoV-2 drugs are exceptionally preliminary and majority of the information available is based on computer-aided findings, for example, using molecular docking and MDs simulation techniques. Detailed *in vitro* and *in vivo* studies utilizing multiple animal models are suggested to gather conclusive evidences before the commencement of clinical studies. A collaborative approach involving marine biologists, chemists, biotechnologists and pharmacists could facilitate and speed up preclinical screening. Obtaining a continuous and consistent supply of sufficient amounts of organisms and compounds without harming the marine environment is another crucial step in the marine drug development process. Possible approaches to counteract this supply problem are the use of biotechnology techniques (aquaculture/fermenter cultivation; genetic engineering; enzymatic synthesis or modification) and by the chemical synthesis/semi-synthesis/modification of isolated bioactive molecules.

**Executive summary**

**Alkaloids**

- Alkaloids obtained from different marine sources have the capacity to bind with and inhibit SARS-CoV-2 main viral protease (M^pro^).

**Polyphenols**

- Docking studies showed that polyphenols play a major role in the deactivation of interaction sites of SARS-CoV-2 proteins that attack human ACE2 receptors in the lung.

**Spirulina**

- A protein-bound pigment of the blue-green algae is commonly used as a food supplement and has immune boosting, anti-inflammatory and SARS-CoV-2 nsp binding properties.

**Marine medicinal agents in clinical trials**

- Griffithsin and plitidepsin have shown promising results in preliminary clinical trials and are actively being investigated for their anti-SARS-CoV-2 potentials.
